# A Cell Biological Perspective on Past, Present and Future Investigations of the Spindle Assembly Checkpoint

**DOI:** 10.3390/biology5040044

**Published:** 2016-11-19

**Authors:** Ajit P. Joglekar

**Affiliations:** Cell & Developmental Biology, University of Michigan Medical School, Ann Arbor, MI 48109, USA; ajitj@umich.edu; Tel.: +1-734-764-2474; Fax: +1-734-615-8500

**Keywords:** mitosis, spindle assembly checkpoint, signal transduction, aneuploidy

## Abstract

The spindle assembly checkpoint (SAC) is a quality control mechanism that ensures accurate chromosome segregation during cell division. It consists of a mechanochemical signal transduction mechanism that senses the attachment of chromosomes to the spindle, and a signaling cascade that inhibits cell division if one or more chromosomes are not attached. Extensive investigations of both these component systems of the SAC have synthesized a comprehensive understanding of the underlying molecular mechanisms. This review recounts the milestone results that elucidated the SAC, compiles a simple model of the complex molecular machinery underlying the SAC, and highlights poorly understood facets of the biochemical design and cell biological operation of the SAC that will drive research forward in the near future.

## 1. Introduction

The primary objective of mitosis is to create two cells with identical genomes. To achieve this, the dividing cell must commence the process of cell division only after every chromosome is stably attached to spindle microtubules emanating from opposite spindle poles. If cell division occurs in the presence of unattached kinetochores, then the result is either chromosome missegregation or loss, and the creation of genetically abnormal, aneuploid cells. To avoid this fate, the dividing cell enforces the requirement for stable kinetochore-microtubule attachment using a cell cycle control known as the spindle assembly checkpoint (SAC). The SAC is a mechanosensitive signaling cascade that ties the progress of the cell cycle machinery with the mechanics of kinetochore biorientation. It is activated by unattached kinetochores, which recruit many different SAC proteins and generate the inhibitory “wait-anaphase” signal. Once the last unattached kinetochore attaches to spindle microtubules, the “wait-anaphase” signal rapidly dissipates, and anaphase ensues. This simple “on-off” operation of the SAC belies an intricate interplay between complex signal transduction machinery embedded in the kinetochore and an equally complex signaling cascade that involves kinases, phosphatases, and numerous SAC signaling proteins. A tight coupling between the signal transduction machinery and signaling cascade of the SAC is essential to minimize chromosome loss and maintain genome stability during cell division.

Understanding the elegant design of the SAC requires an in-depth understanding of both of its component systems: the kinetochore-based mechanochemical signal transduction mechanism that senses the absence of microtubule attachment and the signaling cascade that amplifies and spreads the anaphase-inhibitory signal through the entire cell. This understanding is necessary, because the misregulation of either system can have dire consequences on the genetic stability and health of both daughter cells. Aberrant expression of kinetochore and signaling proteins involved in the SAC are strongly correlated with tumorigenesis and cancer. However, whether and how the aberrant expression directly leads to tumorigenesis is not known. A mechanistic understanding of the kinetochore-based machinery and a quantitative understanding of the SAC signaling cascade can elucidate the causal links that likely connect aberrant SAC function, chromosome missegregation, and tumorigenesis.

This review considers the molecular mechanisms underlying the SAC and their operation from the perspective of cell biology. Extensive molecular, structural, and biochemical investigations of the SAC over the last two decades have achieved a nearly complete description of its signaling cascade, and they elucidate how the kinetochore controls this cascade. Therefore, the following goals were set for this review. The first goal is to briefly summarize the conceptual leaps achieved in understanding the SAC. This summary will highlight studies that deeply influenced the mitosis field, and which continue to guide investigations of the SAC today. The summary uses logical rather than chronological linkage. The second goal is to synthesize a succinct working model for the operation of the SAC. Many expert reviews that delve into the structural details of the SAC signaling proteins and the biochemistry of the SAC were recently published [1,2]. Therefore, this knowledge will be organized in the context of cell biology, so that it is easy to grasp even for readers outside the field of cell division. The final goal for this review is to discuss the major gaps in our understanding of the SAC, and pose four broad questions that are likely to drive future investigations into the SAC.

## 2. Early Hints of a Pathway that Monitors Chromosome Alignment and Controls Anaphase Onset

The foundation for our current understanding of the SAC was established by cell biological investigations conducted almost sixty years ago. For this, the adoption of cine-microscopy proved to be the enabling development. With cine-microscopy, the complete sequence of mitotic events could be documented in real-time for the first time. These observations revealed that the alignment of chromosomes at the metaphase plate is important for the timely onset of anaphase. In one study in particular, Bajer and Mole-Bajer describe the importance of chromosome alignment quite succinctly [3]: “In some cases anaphase does not begin, but ‘waits’ for the chromosome to move to the plate, beginning a few minutes after this has reached it.” An even stronger correlation between chromosome misalignment and delayed anaphase emerged from observations of the first meiotic division in mantid spermatocytes [4]. Mantid spermatocytes contain three sex chromosomes: X1, X2, and Y. Normally, the two X chromosomes pair with the Y to form the sex trivalent. However, if this pairing of sex chromosomes is not successful, the sex chromosomes are unable to align at the metaphase plate. The presence of such misaligned sex chromosomes block cell division for long periods of time. The causative link between chromosome alignment and anaphase onset was made apparent by Zirkle’s experiments [5]. Zirkle used a UV laser micro-beam to irradiate the cytoplasm in the vicinity of metaphase spindles. Laser irradiation damaged the spindle, and dislodged chromosomes from the metaphase plate. Strikingly, the dividing cell containing the misaligned chromosomes remained in mitosis, and initiated anaphase only after these chromosomes realigned at the metaphase plate. Although these results did not implicate unattached kinetochores as the reason for the cell cycle block, they established that the metazoan cell waits until all chromosomes are aligned at the spindle equator before initiating anaphase.

In this context, it is necessary to discuss the truly innovative experiments conducted by Nicklas [6]. Although Nicklas’ experiments were designed to investigate chromosome movement and alignment, their findings deeply influenced our conceptualization of the mechanism of SAC signaling. Using a glass microneedle, Nicklas directly pushed, pulled, and prodded aligned chromosomes during the first meiosis in grasshopper spermatocytes to observe the establishment of bipolar attachments (he referred to this process as “chromosome reorientation”). He noted that the spermatocytes never entered anaphase in the presence of unattached chromosomes. More importantly, he demonstrated that kinetochore-microtubule attachments are stabilized by the application of an opposing mechanical force. The finding that mechanical forces arising from kinetochore interactions with the spindle alter the biochemistry within the kinetochore would provide the basis for the hypothesis that the mechanical force generated by kinetochore-microtubule attachment silences the SAC [7].

Such careful observations of dividing cells derived from diverse organisms provided ample and strong evidence of a system that monitors the alignment of chromosomes at the metaphase plate, and that delays anaphase onset if this alignment is not achieved. However, the significance of these observations remained unclear for nearly three decades, because the biochemical basis of the cell cycle and the concept of cell cycle control were not yet fully understood.

## 3. Discovery of the Spindle Assembly Checkpoint

Until the early 1970s, the cell cycle was viewed as a prescribed sequence of activities and events that proliferating eukaryotic cells progress through [8,9]. According to this view, the completion of each step in the cell cycle sequence was required for and followed by the next step; feedback mechanisms that enforce quality control were not envisioned. Several studies in the 1970s and 1980s challenged this view. Genetic studies discovered many genes, which when mutated, arrested cells in specific stages of the cell cycle [10,11]. Clearly, specific functions encoded by specific genes were essential for the completion of each cell cycle stage. At the same time, biochemical investigations of the synchronous cell divisions that occur during early embryonic development revealed that specific biochemical activities had to be stimulated and then silenced to drive the cell cycle [12]. These findings forced a reconsideration of the nature of the cell cycle. Whether the cell cycle is a set of sequential processes, or if control mechanisms monitor the completion of each process and prevent further progress in case the previous process is not satisfactorily accomplished, became a fundamental question that needed to be addressed.

The existence of feedback mechanisms controlling the progression of the cell cycle was first confirmed by the ground-breaking study by Weinert and Hartwell [13]. The authors screened for genes that are necessary for the cell cycle arrest induced by damaged DNA. They discovered *RAD9* (wild-type gene names appear in upper case italics, mutant genes appear in lower case italics, protein names appear in Roman letters), a gene that is dispensable for the normal cell cycle progression but essential for the cell cycle arrest induced by DNA damage. The discovery of *RAD9* revealed that not only does the eukaryotic cell encode genes that drive cell cycle progression, but also genes that actively monitor the cell cycle, and prevent progress if a crucial step is not satisfactorily completed. This seminal discovery demonstrated that the cell employs feedback controls or checkpoints that monitor the progress of at least one cell cycle phase in order to maintain the quality of the genome [13,14].

The discovery of the DNA damage checkpoint prompted a reconsideration of the early cell biological observations of cell division arrest induced by unaligned and/or unattached chromosomes. Biophysical and biochemical studies of tubulin and microtubules had discovered a number of small molecules that depolymerize microtubules, and thus act as spindle poisons. It was also known that treatment of eukaryotic cells with spindle poisons not only destroys the spindle structure, but also arrests the cells in mitosis [15,16,17]. Furthermore, experimental disruption of kinetochore assembly created chromosomes that could not stably attach to the spindle, and cells containing such unattached or weakly attached chromosomes delayed anaphase for several hours [18]. Could this cell cycle arrest seen in cells with spindle damage also be instituted by another checkpoint? In 1991, two seminal studies discovered two sets of genes that are necessary for arresting cells in mitosis in the presence of microtubule poisons [19,20]. Following the conceptual framework established by the DNA damage checkpoint, these studies hypothesized that specific genes are necessary to institute the metaphase arrest triggered by spindle damage, and therefore, the mutation of these genes will allow cells to enter anaphase even though the spindle is damaged and chromosome segregation is impaired. By screening mutant budding yeast cells that cannot arrest in the presence of spindle damage, they discovered two classes of aptly named genes: mitotic arrest deficient (*MAD*), and budding uninhibited by benzimidazole (*BUB*) genes [19,20]. Because this checkpoint appeared to respond to damage to the mitotic spindle, it was termed the spindle assembly checkpoint. This discovery confirmed that the eukaryotic cell uses a surveillance mechanism to regulate the metaphase to anaphase transition.

## 4. Elucidation of the Design and Operation of the SAC

**Figure 1:**
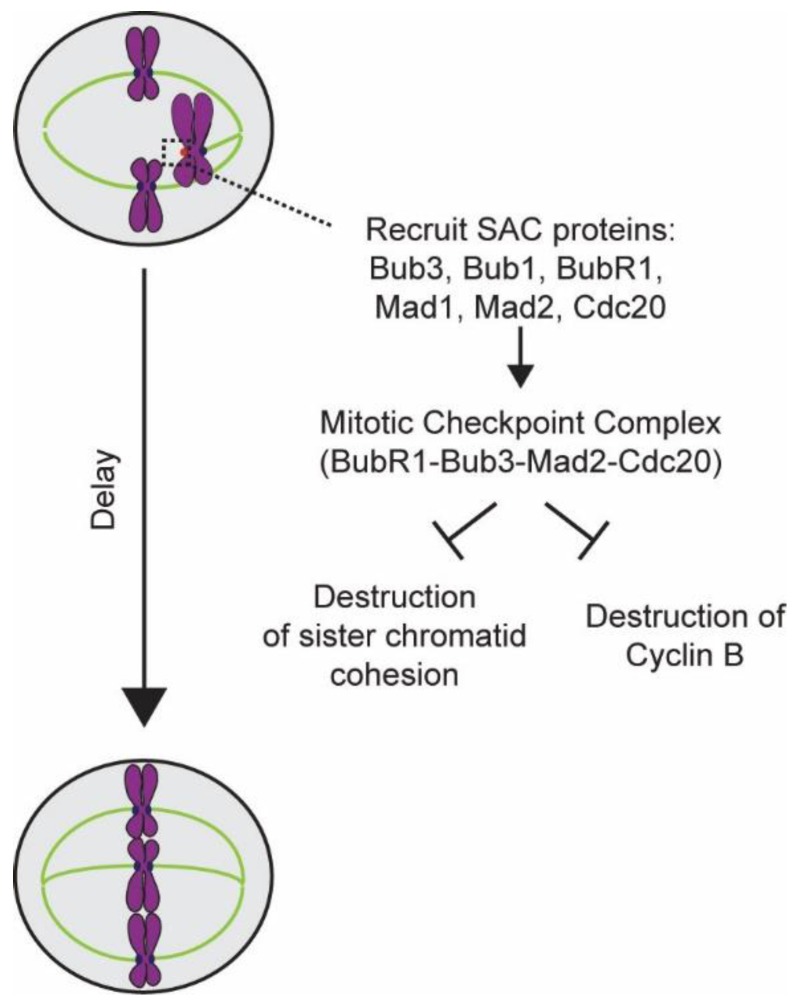
Schematic of the overall design of the spindle assembly checkpoint (SAC).

The discovery of the SAC unleashed a decade-long search for genes and proteins involved in the implementing it. In retrospect, these efforts resemble an exciting game of solving a complex jigsaw puzzle. Important pieces of this puzzle, in the form of major SAC activities, were already in hand. It was known that the SAC detects spindle damage and then blocks the onset of anaphase in response (Figure 1). To be able to accomplish these functions, the SAC must perform at least three activities: (1) detect damage to the spindle and/or unaligned chromosomes in the spindle, (2) inhibit anaphase onset, and (3) prevent sister chromatid separation. With this knowledge, the race to solve the SAC jigsaw puzzle was underway.

As discussed earlier, the significance of chromosome attachment to the spindle for timely anaphase onset was clear from early cytological studies. Therefore, cell biologists suspected that the mitotic arrest observed upon spindle damage was a response to the creation of unattached chromosomes rather than damage to the spindle. To explain why cells with damaged spindles arrest in mitosis, McIntosh presented a clear, mechanistic hypothesis [7]. He proposed that the centromeric region of unattached chromosomes generates a “wait-anaphase” signal in order to prevent anaphase onset. Influenced by Nicklas’ vivid demonstration of the ability of mechanical forces to influence the biochemistry of kinetochore-microtubule attachment, McIntosh also proposed that the tension in the centromeric region of each chromosome, which is generated by the opposing forces generated by sister kinetochores, plays a critical role: it stops the production of the “wait-anaphase” signal, and thus silences the SAC. Evidence in support of McIntosh’s hypotheses accumulated quickly through cell biological experimentation. For example, mutations in the DNA sequence of the genetically defined point centromere found in budding yeast significantly delayed mitosis [21]. This result independently confirmed the correlation between centromere function and cell cycle progression that had been established by the Earnshaw group [22]. Rieder’s classic experiment involving laser ablation provided unequivocal evidence for the activating role of unattached kinetochores in SAC [23,24]. Rieder showed that cells containing even one unattached kinetochore were blocked in metaphase. Importantly, he showed that the ablation of this single unattached kinetochore by a focused laser beam was sufficient to remove the metaphase block, and allow the cell to enter anaphase within minutes. This result confirmed that the kinetochore detects a lack of microtubule attachment, and transduces this information into a biochemical signal to prevent anaphase onset.

How does the unattached kinetochore transduce information regarding the lack of microtubule attachment and convey it to the biochemical machinery that drives the cell cycle? Elucidation of the biochemical activities involved in this signal transduction process took place at a rapid pace, because the genes involved in SAC signaling were already known. The discovery of *MAD2* led to the characterization of the function of its product, Mad2, in human cells and in *Xenopus* extracts [25,26]. This work confirmed that Mad2 is necessary for activating the SAC, and more importantly, demonstrated that Mad2 localizes exclusively at unattached kinetochores. These findings revealed that unattached kinetochores are also the site of the biochemical activity that generates the “wait-anaphase” signal. Characterization of the Mad2 protein also led to the discovery of its binding partner, the protein Mad1 [27]. Subsequent studies localized other SAC proteins to unattached kinetochores as well, and found that this localization is highly dynamic [28,29]. The dynamic nature of SAC protein localization lent credence to the notion that kinetochores assemble the “wait-anaphase” signal, which then spreads throughout the cell volume to inhibit anaphase onset.

After unattached kinetochores were established as the site of SAC signal generation, the focus of research turned to the mechanism by which the SAC signal prevents anaphase onset. Vital clues to this puzzle were already in hand: that cyclin-dependent kinase 1 regulates mitosis, that its activator, cyclin B, is degraded in anaphase, and that this degradation occurs via a ubiquitin-mediated pathway [30,31,32]. Clever genetic screens and biochemical experiments based on these clues led researchers to subunits of the anaphase promoting complex/cyclosome (APC/C) [33,34]. With the discovery of the APC/C, researchers focused their attention on how the cell inhibits APC/C prior to anaphase. Using genetic screens of mutant alleles of genes implicated in the cell division cycle (*CDC* genes), two studies discovered the activating subunits of the APC/C: Cdc20 and Cdh1 [35,36]. The discovery of Cdc20 shifted research focus to the biochemical nature of the kinetochore generated “wait-anaphase” signal. These investigations found that the SAC proteins Bub3, Mad2, Mad3/BubR1, and Cdc20 interact with one another, and that this interaction is necessary to sequester Cdc20 [37,38]. The complex of these four proteins came to be known as the Mitotic Checkpoint Complex (MCC). Careful biochemical characterization revealed that formation of the MCC depletes Cdc20 from the cytosol, and thus deprives APC/C of its activating subunit. Thus it was finally clear that unattached kinetochores activate the SAC by recruiting SAC proteins, and enabling them to bind to and sequester Cdc20. Sequestration of Cdc20 keeps APC/C inactive and inhibits anaphase onset. As discussed later, a very recent study demonstrates that the MCC uses yet another mechanism in order to act as a potent inhibitor of the APC/C [39].

In addition to preventing anaphase onset, the SAC must also protect the cohesion between sister chromatids during the cell cycle arrest. This notion was supported by the observation that cells carrying mutations in the APC/C genes not only arrested in mitosis, but also failed to separate sister chromatids [40,41]. A genetic screen based on this observation yielded the precocious dissociation of sister chromatids gene (*PDS1*), and revealed that mutations in *PDS1* led to the premature separation of sister chromatids prior to anaphase. Furthermore, this observation suggested that the APC/C might target a protein involved in sister chromatid cohesion for degradation. In fact, earlier studies had shown that complete degradation of cyclin B, the main APC/C target known at the time, is not necessary for anaphase onset [42]. Using the discovery of *PDS1* as a toe-hold, researchers designed yet another genetic screen that yielded subunits of the Cohesin complex, a remarkable protein clamp that hold sister chromatids together, and which must be broken apart by APC/C-directed proteolysis to allow sister chromatid separation [43,44]. Discoveries of *PDS1* and the Cohesin complex clarified the role of Pds1 as the inhibitor of Cohesin destruction [45,46]. With this information, the protease that cleaves Cohesin, known as separase, was also discovered [47,48]. These discoveries outlined the third process that is necessary for the effective operation of the SAC.

In this manner, the major pieces of the jigsaw puzzle of the SAC were set in place in less than 10 years after its discovery. This knowledge led to the discovery of new proteins and activities critical to SAC function. Of note, the kinetochore proteins Ndc80 and the Spc105 were identified [49]. These proteins were later revealed to be critical components of the SAC activation machinery. The involvement of Mps1 kinase in SAC signaling was also revealed [50]. Thus, a firm foundation for defining the molecular components and biochemical activities of the SAC was established.

## 5. Molecular Mechanisms Underlying SAC Activation and Inactivation

The operation of the SAC during cell division is deceptively simple. Each unattached kinetochore generates a biochemical signal to inhibit APC/C activity, and thereby preventing the degradation of mitotic proteins and sister chromatid cohesion. Once the last unattached kinetochore forms a stable attachment to the mitotic spindle, the APC/C is unleashed and anaphase ensues. This seemingly simple sequence of events requires the interlocked operation of three distinct processes, each of which employs the coordinated activity of many different proteins. These processes are: (1) detection of the lack of attachment by the kinetochores, (2) recruitment of SAC proteins to the unattached kinetochore and production of the wait-anaphase signal, and (3) rapid inactivation of the “wait-anaphase” signal after the last kinetochore forms stable attachment to the spindle. The following discussion presents a concise description of the current understanding of the mechanisms underlying these individual processes.

### 5.1. Detection of the Lack of “End-On” Microtubule Attachment to the Kinetochore

During cell division, the eukaryotic kinetochore grabs onto an approximately 40 nm section of the plus-ends of one or more microtubules even as these plus-ends grow and shrink [51,52]. The kinetochore is exquisitely sensitive to such “end-on” attachment: it is able to distinguish this type of attachment from the absence of microtubule attachment as well as from the so-called “lateral” attachment to the microtubule lattice. Moreover, the kinetochore responds to a change in its end-on attachment state almost instantaneously as inferred from the loss or recruitment of SAC proteins by the kinetochore upon the gain or loss of end-on attachment [53,54]. How does the kinetochore detect changes in its attachment state and then transduce this information into a biochemical signal?

**Figure 2:**
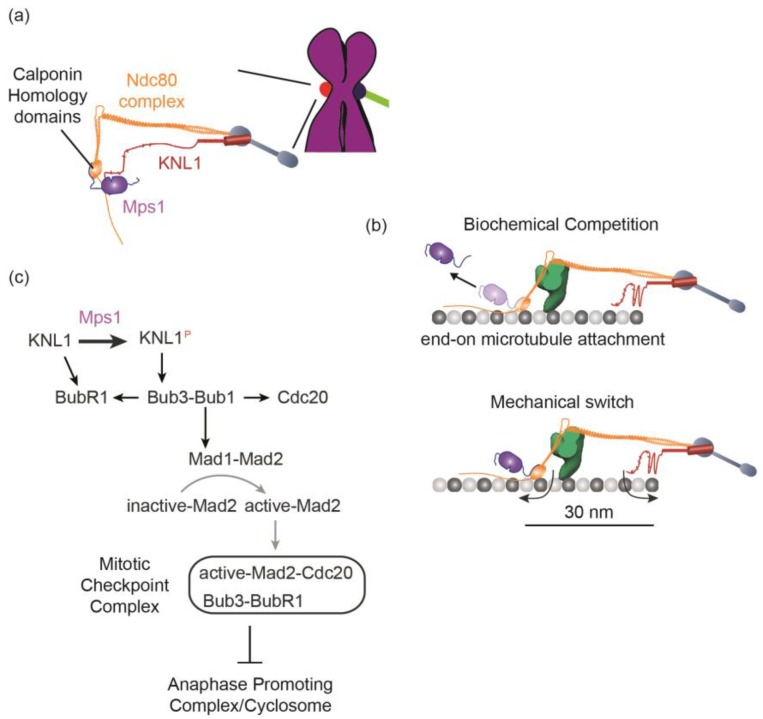
(**a**) Cartoon depicting the three-component microtubule-sensing mechanism in a kinetochore that lacks end-on microtubule attachment. (**b**) Microtubule attachment disrupts SAC signaling via two distinct mechanisms. The cartoons depict 1D visualization of the budding yeast kinetochore [66]. The green shape represents the Dam1 ring found in budding yeast; the blue rod like molecule is the Mtw1/Mis12 complex. Top: end-on attachment dislodges a large fraction of the Mps1 from the kinetochore. Bottom: End-on attachment separates the Calponin-Homology domains and the phosphodomain of Spc105/KNL1 from each other. (**c**) A simplified schematic of the biochemical interaction network that recruits SAC proteins to the unattached kinetochore. Black arrows represent binding to proteins localized in the kinetochore. Gray arrow indicates a conformational change that converts inactive Mad2 (also known as “Open” Mad2) into its active form (“Closed” Mad2).

The kinetochore relies on two properties to control the SAC: the biochemical activities of a trio of protein components and their nanoscale organization in the kinetochore (Figure 2a). The three protein components are the kinetochore protein complexes Ndc80 and Spc105/KNL1-ZWINT1 and the Mps1 kinase. The function of each protein is clear. The Ndc80 complex is the essential recruitment site for Mps1 in the kinetochore, while Spc105/KNL1 is the primary target of Mps1 kinase activity [55,56,57,58]. Mps1 phosphorylates Spc105/KNL1 at several phosphorylation sites to start a biochemical cascade that recruits all the SAC proteins, including Mad2, to the kinetochore. Importantly, the phosphorylation of Spc105/KNL1 by Mps1 is exquisitely sensitive to end-on microtubule attachment. The sensitivity stems from the ingenious design of the end-on kinetochore-microtubule attachment. End-on attachment is established by the Calponin Homology domains of the Ndc80 complex, the same domains that recruit Mps1 [59,60]. Moreover, the microtubule-binding and Mps1-binding surfaces in the Calponin Homology domains partially overlap. Consequently, Mps1 directly competes with the microtubule tip for binding the Calponin Homology domains. It robustly binds unattached kinetochores, but gets dislodged during the formation of end-on microtubule attachment (Figure 2b top). This prevents the phosphorylation of Spc105/KNL1, and thus disrupts SAC signaling.

The competition between Mps1 and the microtubule tip for binding to the Calponin Homology domain removes a large fraction of Mps1, but not all of it. Moreover, under certain conditions, kinetochores with end-on attachments retain Mps1 and recruit SAC proteins, but they do not delay anaphase onset [61,62]. Finally, Mps1 kinase activity is present within the metaphase kinetochore possessing end-on microtubule attachment, because it activates the SAC if Mad1 is artificially tethered to the kinetochore [63,64,65]. Why does the residual Mps1 in the metaphase kinetochore not phosphorylate Spc105/KNL1 nor activate the SAC?

The answer to this question is likely to lie in the architecture of the kinetochore-microtubule attachment [66]. High-resolution colocalization of fluorescently labeled kinetochore proteins in budding yeast, *Drosophila*, and human kinetochores revealed that each protein occupies a distinct average position along the long axis of the end-on kinetochore-microtubule attachment [67,68,69]. Importantly, the Calponin Homology domains are separated from the phosphodomain of Spc105/KNL1 by a distance of ~30 nm. This means that the Mps1 bound to the Calponin Homology domains will also be 30 nm away from Spc105/KNL1, its phosphorylation target. This small separation can be crucial for the SAC as demonstrated by the analysis of the budding yeast kinetochore [66]. Like human kinetochores, budding yeast kinetochores retain a fraction of Mps1, even after forming end-on microtubule attachment [55,66]. In budding yeast, the only reason why this residual Mps1 cannot activate the SAC is that the 30 nm separation between the Mps1 binding site in the kinetochore and Spc105/KNL1 in the kinetochore-microtubule attachment prevents Mps1 from phosphorylating Spc105/KNL1. In fact, experimentally bridging the 30 nm gap between the kinase and its substrate, either by moving Mps1 closer to Spc105/KNL1, or vice versa, is sufficient to re-activate the SAC even on kinetochores with stable end-on microtubule attachments. These findings suggest that the yeast kinetochore functions as a mechanical toggle-switch comprising the Calponin Homology domains of the Ndc80 complex and the phosphodomain of Spc105/KNL1. Bringing the two terminals of this toggle-switch close together turns the SAC on, whereas their separation turns the SAC off. Only stable end-on microtubule attachment can reliably and persistently separate the two terminals from one-another (Figure 2b, bottom). Structural properties of the Ndc80 complex, most notably its flexible hinge, likely facilitate the microtubule in pulling the Calponin Homology domain away from the phosphodomain of Spc105/KNL1 [70]. Similarly, the phosphodomain of Spc105/KNL1 also binds to the microtubule, which probably prevents it from approaching the Calponin Homology domains [71]. Thus, the nanoscale architecture of the yeast kinetochore plays an essential role in its ability to detect end-on microtubule attachment.

These investigations elucidate how the eukaryotic kinetochore senses end-on microtubule attachment. Even though the role of kinetochore architecture in sensing microtubule attachment was revealed using the budding yeast kinetochore, this role is likely to pertain to a wide range of organisms because of the remarkable conservation of the architecture of the end-on kinetochore-microtubule attachment. This role also highlights the functional significance of such attachments during mitosis. End-on kinetochore-microtubule attachments have been found in mitotic cells of nearly every eukaryotic organism that has been studied so far [52]. However, such attachments are not necessary for chromosome congression; achievement and maintenance of chromosome congression reflects a balance of opposing forces acting on each chromosome. Indeed, the holocentric chromosomes in *C. elegans* congress to the spindle equator using lateral kinetochore-microtubule attachments during meiosis [72]. Even in HeLa cells, chromosome congression to the metaphase plate can be achieved using lateral attachments, if the force generation mechanisms in the mitotic spindle are suitably manipulated to facilitate this process [73]. Yet, end-on attachment is highly conserved throughout eukaryotic evolution. An obvious reason for this conservation may be that end-on attachment seamlessly integrates the feedback control mechanism and the force generation machinery in the kinetochore [74,75].

### 5.2. Generation of the Mitotic Checkpoint Complex

Unattached kinetochores recruit SAC signaling proteins from the cytosol to generate the “wait-anaphase” signal in the form of the Mitotic Checkpoint Complex (Figure 2c). This recruitment is achieved by a cascade of biochemical interactions that recruit Bub3, Bub1, BubR1, Cdc20, as well as Mad1 and Mad2. This cascade is initiated when Mps1 phosphorylates Spc105/KNL1 [76,77,78]. Mps1 targets several sites within Spc105/KNL1 with the consensus amino acid sequence “Met-Glu-Lys-Thr”, commonly referred to as MELT repeats. Each phosphorylated MELT repeat can bind one molecule of the Bub3-Bub1 protein complex by making contact with residues in both Bub3 and Bub1 [79]. The recruitment of the Bub3-Bub1 complex is the key event, because Bub1 provides a binding interface for BubR1, Cdc20, and the Mad1-Mad2 complex [80,81]. BubR1 also recruits Cdc20 [81]. Finally, the metazoan Spc105/KNL1 protein contains two related sequences known as KI motifs because of their amino acid sequence, each of which binds directly to Bub1 and BubR1 respectively in an Mps1-independent manner [82,83,84]. Bub1 molecules recruited to the kinetochore as part of the Bub3-Bub1 complex are also phosphorylated by Mps1, which enables them to interact with and recruit the heterotetrameric Mad1-Mad2 complex to the kinetochore [80,85]. These regulated biochemical interactions together ensure that all components of the MCC are localized within an unattached kinetochore, and pave the way for MCC formation. However, the Mad1-Mad2 complex recruited to the kinetochore in this manner does not become a part of the MCC. Instead it participates in catalyzing the conversion of “inactive” conformations of cytosolic Mad2 into an “active” conformation, which then gets incorporated into the MCC [86].

The molecular mechanism by which Mad2 switches between active and inactive conformations is yet another fascinating process in the cell cycle, and its molecular details have been the subject of several studies [1]. Suffice it to say here that the Mad2 molecule complexed with Mad1 assumes the active conformation, and it forms a conformational heterodimer with cytosolic Mad2 molecules that are in the inactive conformation, thus recruiting these molecules to the kinetochore [87]. The Mad1-Mad2 complex is then thought to act as a template that converts inactive Mad2 molecules into their active form [86]. Intriguingly, Mps1 kinase activity is also required for the conversion of the inactive Mad2 conformation into the active conformation [88,89]. The functional effect of the Mps1-mediated phosphorylation in the conformational change is unknown. However, the involvement of Mps1 in every step of the SAC cascade suggests that it functions as a “licensing kinase” that firmly tethers the entire signaling cascade to unattached kinetochores.

In addition to SAC protein recruitment, several molecular interactions add to the complexity of the central SAC signaling cascade and ensure robust SAC signaling. The Aurora B kinase, which is involved in the error correction pathway, enhances SAC signaling by promoting Mps1 recruitment [90]. Polo-like kinase 1, which licenses centrosome duplication, also phosphorylates Cdc20 molecules within the kinetochore to prevent them from activating the APC/C [80,91]. The ultimate goal of this network of biochemical interactions is to generate the MCC, which then inhibits APC/C. It is important to note that this simple description does not explain the remarkable potency with which the MCC inhibits the APC/C: one or a few unattached kinetochores generate enough MCC to affect mitotic progression within ~5 minutes [54]. Recently published biochemical experimentation explains why the APC/C is highly sensitive to MCC [39]. As discussed earlier, the MCC reduces APC/C activation by sequestering Cdc20. Additionally, it also binds to a second molecule of Cdc20 that is already complexed with APC/C, and by doing so, inhibits the APC/C that has been activated. Finally, APC/C itself also contributes to the maintenance of the SAC by targeting Cdc20 for degradation [92].

From the perspective of cell biology, the complex biochemical interactions that generate MCC must meet two demands. First, they must ensure that a single unattached kinetochore can produce a sufficiently large quantity of MCC so that anaphase is delayed and chromosome missegregation is averted. Second, they must also ensure that the generation of MCC does not scale linearly with the number of unattached kinetochores in the cell [93]. A dividing cell contains a large number of unattached kinetochores in prophase. If these kinetochores produce a proportionately large quantity of MCC, then the result could be the accumulation of a vast excess of MCC, and consequently, unnecessarily delay in anaphase onset even after all chromosomes attach to the spindle. Meeting these contrasting demands using a biochemical signaling cascade is challenging. In fact, cell biological experimentation suggests that the kinetics of MCC generation and the steady-state MCC concentration can fall short of the target necessary for complete APC/C inhibition. If this happens and APC/C activity is not fully inhibited, the residual activity steadily degrades cyclin B, and perhaps other mitotic proteins, until cyclin B levels fall below the threshold necessary to maintain the biochemical state of the cell corresponding to mitosis [94]. As a result, the cell enters anaphase even as it contains unattached kinetochores [95,96].

The significance of the kinetics of MCC generation and steady-state concentration of MCC was demonstrated by a set of three elegant studies. One of these studies, which was discussed earlier, created different numbers of unattached kinetochores by destroying their attachment to the spindle in metaphase cells, and asked whether these cells activated the SAC and arrested in mitosis [54]. This study found that the unattached kinetochores do not always inhibit anaphase. Despite recruiting normal levels of SAC proteins, these kinetochores cannot fully suppress APC/C activity presumably because they cannot produce a sufficient quantity of MCC. This observation implies that the signaling cascade of the SAC must be calibrated such that a single unattached kinetochore produces a sufficiently large signal at a high rate. Two other studies, one in fission yeast and the other in human cells, demonstrated that the duration of the SAC-mediated metaphase arrest inversely correlates with the rate of cyclin B degradation. Therefore, the steady-state MCC concentration also directly correlates with the duration of the mitotic arrest achieved [97,98]. The functional significance of the kinetics of MCC generation and steady-state MCC concentration in the dividing cell is also apparent from the results of two unrelated studies. In human cells, MCC generation begins in interphase via interactions of the Mad1-Mad2 complex with the Mps1 kinase at the nuclear envelop [99]. Although the exact mechanism of MCC generation in this case is unclear, this pool of MCC is required for accurate chromosome segregation. This surprising result suggests that the MCC generated during interphase inhibits APC/C activity during early mitosis to slow down mitotic progression, while the kinetochore-based MCC generation cascade is ramping up. Thus, the interphase MCC generation effectively acts as a buffering mechanism to minimize APC/C activity during early mitosis. Finally, the significance of the MCC generation capacity of the kinetochore was also demonstrated by observations of SAC signaling in *Xenopus* egg extracts. These experiments revealed that kinetochores expand their signaling capacity in order to bolster the steady-state MCC concentration [100].

### 5.3. Inactivation of the SAC

SAC inactivation is mediated by two distinct processes. The first process involves the silencing of the SAC signaling events within a kinetochore. The kinetochore loses SAC proteins within a couple of minutes after establishing end-on microtubule attachments. Critical to this process is the disruption phosphorylation of Spc105/KNL1, and potentially Bub1 and Mad1, by end-on microtubule attachment as discussed earlier. This event enables Protein Phosphatase 1 (PP1) to remove the phosphorylation on Spc105/KNL1. In fact, KNL1/Spc105 uses a conserved PP1 recruitment motif within its phosphodomain to recruit PP1 to the kinetochore [101,102]. Moreover, recent work shows that Protein Phosphatase 2A (PP2-B56) recruited by the kinetochore-bound BubR1 also dephosphorylates Spc105/KNL1 [103]. Finally, a microtubule-binding component of the metazoan kinetochore, the spindle and kinetochore-associated (SKA) complex, was also shown to recruit PP1 and promote SAC silencing [104]. Additionally, metazoan kinetochores employ dynein motors, which strip kinetochore-bound Mad1-Mad2 complexes and carry them to the spindle poles along the kinetochore-attached microtubules [53]. These processes together ensure that kinetochores with end-on microtubule attachments do not signal.

The second process of SAC inactivation rapidly dissipates the checkpoint signal in the cytosol. Metazoan cells employ two mechanisms to achieve this. A protein known as p31comet uses a particularly fascinating mechanism. p31comet structurally mimics the active conformation of Mad2 [105]. This allows it to bind to MCC, and extract Cdc20 from it. Additionally, a specialized ATPase called Thyroid receptor hormone interacting protein (TRIP13) converts the active form of Mad2 into the inactive form [106,107,108]. Both mechanisms are operational throughout mitosis, not just in anaphase. They ensure that anaphase ensues without delay after the last unattached kinetochore forms stable attachment.

The process of SAC silencing is usually rapid, as evidenced by live-cell observations that show that anaphase onset takes place within 15 minutes after the last unattached kinetochore forms end-on attachments [24]. Unnecessary delay in anaphase onset, even after all chromosomes have established bipolar attachment, is unlikely to have any positive outcomes. In fact, prolonged mitotic arrest is often deleterious to the cell [95]. A large fraction of the cells that arrest in mitosis undergo apoptosis. They also suffer from “cohesion fatigue” due to the degradation of sister chromatid cohesion over time [109,110,111,112]. Prolonged mitosis can also alter cell fate in the tissue context [113]. The mechanisms discussed above are likely crucial for avoiding these negative outcomes.

## 6. Directions for Future Investigations of the SAC

The extensive research spanning over two decades affords us a deep, mechanistic understanding of many facets of the SAC. This understanding provides a solid foundation to attack areas of the SAC that are not well-understood. The discussion below highlights four such areas.

### 6.1. What Does the Kinetochore Respond to—End-On Attachment to the Kinetochore, an Architectural Change within the Kinetochore Induced by Such Attachment, or Both?

This topic has been the subject of debate for many years [114,115]. As discussed in the previous section, two different mechanisms have been shown to disrupt SAC signaling at the kinetochore: biochemical competition and attachment-induced separation of two protein domains. Although it is clear that the physical separation of the Mps1 kinase bound to the Calponin-Homology domains from the Spc105/LNL1 phosphodomain is essential for SAC silencing in budding yeast, whether this mechanism is also important for SAC silencing in metazoan kinetochores must be addressed. In comparison to the budding yeast kinetochore, kinetochores in most other eukaryotes offer an additional challenge to SAC silencing. These kinetochores typically bind the plus-ends of many microtubules unlike the budding yeast kinetochore, which binds just one microtubule [52]. Furthermore, these microtubule attachments are dynamic: old attachments are lost and new attachments form even in metaphase. This means that the metazoan kinetochore will contain a number of unbound Calponin Homology domains. Why don’t these domains recruit Mps1 and activate the SAC? Neither the biochemical competition model nor the mechanical switch model offers a satisfactory explanation. It is likely that additional mechanisms suppress signaling activity from metaphase kinetochores. Alternatively, it is possible that metaphase kinetochores may harbor trace SAC signaling activity that is not detectable by conventional methods.

It is also important to note here that the attachment-induced separation of two kinetochore proteins from one-another has sometimes been construed as “intra-kinetochore stretch”, and hence considered a tension-based mechanism [116,117,118]. However, physical separation of two mechanically unlinked protein domains does not necessarily require a large force. The Ndc80 complex is linked to Spc105/KNL1 on the centromeric ends of the respective molecules. Crucially, however, the Calponin Homology domains and the unstructured phosphodomain of Spc105/KNL1 are not linked to each other, and as such they are likely to be free to move within a certain radius about the linked, centromeric ends of the two protein molecules. Therefore, sustained separation of their free ends can be achieved by the maintenance of the architecture of the end-on kinetochore-microtubule attachment. If this hypothesis is true, then it creates the possibility that end-on attachment is necessary, but not sufficient, to silence the SAC.

### 6.2. How Does the Kinetochore Generate a Sufficiently Large Quantity of MCC at a High Rate?

The kinetochore contains a rather small number of molecules of Ndc80 and Spc105/KNL1. For example, a human kinetochore contains approximately 250 molecules, whereas the kinetochore in the much smaller budding yeast contains only 8 molecules of Ndc80 and Spc105/KNL1 [119,120,121]. Yet, this small number of molecules is capable of generating a sufficiently large quantity of MCC, and delay cell division. In this regard, it is interesting that each Spc105/KNL1 molecule contains a large number of MELT motifs: ~19 in human cells [78]. Since each MELT motif can bind one Bub3-Bub1 complex, 19 MELT motifs, in principle, should be able to bind 19 Bub3-Bub1 molecules, and generate a 19-fold higher SAC signal. However, careful analysis of Bub3 recruitment reveals that on average only 30% of the MELT motifs bind Bub3-Bub1 [93,122]. In fact, engineered KNL1 molecules with just a single MELT motif and the KI repeats suffice to activate the SAC when cells are treated with nocodazole [82]. Why Spc105/KNL1 contains many MELT motifs but uses only a small fraction of these motifs, and how it succeeds in generating a strong wait-anaphase signal, are fundamental questions at the heart of the SAC. A significant challenge in addressing these questions is that the biochemical reactions leading up to the generation of the MCC take place in the nanoscopic structure of the kinetochore. The crowded environment of the kinetochore makes it extremely difficult to measure the biochemical rates of individual reactions. Whether and how the localization of SAC proteins within unattached kinetochores alters or enhances the rate of MCC generation is also a key question that needs to be addressed.

### 6.3. Is the SAC a Switch or a Rheostat?

This complex question does not have a simple answer. The operation of the SAC during cell division gives the distinct impression of a switch-like behavior [4,23,24]. The response of the kinetochore to end-on microtubule attachment is also switch-like. On the other hand, the SAC can have different strengths specified by different the steady-state level of MCC in the dividing cell. Consequently, an active SAC can produce different lengths of delay in anaphase onset [97,123]. This SAC behavior is analogous to that of a rheostat. It is also worth noting that a kinetochore can be actively generating MCC, but unable to inhibit anaphase [54]. Therefore, analysis of the SAC that takes into account the two separate systems that underlie its operation: the kinetochore-based SAC activation system and the cytoplasmic SAC signaling cascade, is needed to define the operation of the SAC in its entirety.

### 6.4. Can a Defective SAC Cause Aneuploidy?

The majority of tumors contain aneuploid cells, and exhibit high rates of chromosome missegregation. Because of the critical role that the SAC plays in ensuring accurate chromosome segregation, aberrant SAC signaling is likely to be an essential aspect of cancer cell biology. In fact, cancer cells quite frequently misregulate the expression of one or more critical SAC protein including Mad2, Bub3, Bub1, and BubR1 [124]. The strongest data implicating aberrant SAC come from studies of mouse models [125]. However, whether aberrant expression of SAC proteins is directly responsible for generating aneuploidy that leads to tumorigenesis and cancer is not clear. This is because many cancer cell lines appear to have a functional SAC, even if they express SAC proteins aberrantly [126]. Therefore, whether an aberrant SAC is the causative factor of aneuploidy and tumorigenesis, or if it is a consequence of aneuploidy arising from other factors, needs to be determined.

The uncertainty regarding the role of the SAC in cancer cell biology likely stems from the fact that the SAC is a biochemical approximation of a toggle-switch. Despite its switch like operation, the strength of the SAC depends on the steady-state level of MCC generation and the rate of MCC generation by individual kinetochores [54,97,98]. This means that the conventional methodology for assessing SAC function, which is to depolymerize the spindle and quantify the duration of cell cycle arrest, suffers from a key limitation. This method quantitates only the maximum strength of the SAC. It cannot determine whether and how subtle changes in SAC strength, i.e., the potency of MCC generation, caused by aberrant expression of one or more SAC proteins affect chromosome segregation accuracy. This is because conventional assays based on spindle depolymerization generate a large number of unattached kinetochores, and thus mimic the prophase, when the dividing cell contains a large number of unattached kinetochores. As mitosis progresses, unattached kinetochores attach to spindle microtubules and cease to signal, and finally just one unattached signaling kinetochore is left. This is when optimal strength of the SAC is the most critical. Only if the last unattached kinetochore reliably delays cell division, chromosome missegregation will be averted. The misregulation of SAC genes can impact the ability of this kinetochore to delay cell division, and hence increase the rate of chromosomal instability. Future studies of the SAC will require new techniques to quantify subtle changes in the SAC signaling cascade, and then study whether such changes elevate the rate of chromosome missegregation during cell division.

## 7. Conclusions

Research spanning over two decades has revealed the elegant biochemical design, the molecular complexity, and the efficient cell biological operation of the SAC. On-going innovative research is adding new dimensions to the SAC field. For example, a truly fascinating field is the evolutionary biology SAC genes and proteins [127,128]. Scaling of SAC strength with changing cell size during development is another topic that merits attention [129]. These investigations will add to this knowledge, and fully define the molecular mechanisms and design of the SAC.

A pressing need for enabling a complete understanding the SAC is the technical capability to experimentally control it in vivo, and then quantify the individual biochemical reactions in the SAC signaling cascade. Mathematical models to simulate the operation of SAC in space and time are also necessary. Only such models can account for how the operation of the SAC changes in the context of a number of parameters, biochemical (concentrations of SAC proteins), physical (kinetochore size and the volume of the dividing cell), and physiological (species specific duration of the cell cycle, number of chromosomes, etc.), that characterize the dividing cell [96,130,131]. Integration of quantitative data with mathematical modeling will likely elucidate the biochemical design and cell biological operation of the SAC.
